# Dissecting Molecular Genetic Mechanisms of 1q21.1 CNV in Neuropsychiatric Disorders

**DOI:** 10.3390/ijms22115811

**Published:** 2021-05-28

**Authors:** Joy Yoon, Yingwei Mao

**Affiliations:** Department of Biology, Eberly College of Science, Pennsylvania State University, University Park, PA 16802, USA; jfy5125@psu.edu

**Keywords:** copy number variation, microdeletion, microduplication, schizophrenia, autism spectrum disorder, microcephaly, macrocephaly, neurodegeneration, synaptic plasticity

## Abstract

Pathogenic copy number variations (CNVs) contribute to the etiology of neurodevelopmental/neuropsychiatric disorders (NDs). Increased CNV burden has been found to be critically involved in NDs compared with controls in clinical studies. The 1q21.1 CNVs, rare and large chromosomal microduplications and microdeletions, are detected in many patients with NDs. Phenotypes of duplication and deletion appear at the two ends of the spectrum. Microdeletions are predominant in individuals with schizophrenia (SCZ) and microcephaly, whereas microduplications are predominant in individuals with autism spectrum disorder (ASD) and macrocephaly. However, its complexity hinders the discovery of molecular pathways and phenotypic networks. In this review, we summarize the recent genome-wide association studies (GWASs) that have identified candidate genes positively correlated with 1q21.1 CNVs, which are likely to contribute to abnormal phenotypes in carriers. We discuss the clinical data implicated in the 1q21.1 genetic structure that is strongly associated with neurodevelopmental dysfunctions like cognitive impairment and reduced synaptic plasticity. We further present variations reported in the phenotypic severity, genomic penetrance and inheritance.

## 1. Introduction

Rare CNVs, such as chromosomal deletions and duplications, have raised much scientific interest in etiological studies of NDs. It has been suggested that genetics play a major role in NDs, with ~52.4% and ~80% of inheritability in ASD and SCZ, respectively. A genetic study has shown that rare and large CNVs are likely to be causative, as they can lead to numerous gene imbalances [1]. Case–control studies have demonstrated that rare CNVs occur at higher frequency in cases than in controls, suggesting that patients bear a high CNV burden [2,3]. Moreover, 17.1% of those who presented abnormal clinical presentations carried pathogenic CNVs [4]. Approximately 40% of carriers had de novo mutations, and the majority of the de novo mutations (91%) were pathogenic [4]. These patterns show up in most ND studies, including ASD, SCZ, intellectual disability (ID) and attention deficit hyperactivity disorder (ADHD) [5,6,7]. These findings shed light on the contribution of CNVs to the risks of different NDs.

In general, CNVs are pleiotropic and have variable expressivity, in that different patients carrying CNVs at the same chromosomal regions can show the symptoms of different psychiatric disorders; for example, many ASD-associated CNVs are also found in SCZ patients [3,4,8,9]. Despite having the same CNV carriers, phenotypes and severity range diversely, and show incomplete penetrance [10]. This suggests that there must be other factors involved, such as other genetic components (the two-hit model) [11] or environmental factors [12]. Hence the complexity of CNVs has been underscored in the etiology of ND.

A recent GWAS has identified risk loci prevalent in NDs, which are rare CNVs seen in cases but not in controls [2]. At least eight distinct CNVs,1q21.1, 2p16.3, 3q29, 7q11.23, 15q13.2, 16p11.2, 22q11.2 and NRXN1, have been consistently reported as risk factors for many NDs [6,7,8,13,14,15]. Deletions are less frequent but more pathogenic than duplications. Therefore, an increased odds ratio (OR) was found for deletions (i.e., ORs of 1q21.1 = 11.82 (del) and = 6 (dup)) [15]. The abnormal clinical presentations are postulated to be a result of carrying those pathogenic CNVs. Many genetic studies have attempted to identify the relationships between genetic rearrangements in the regions and clinical phenotypes. As little is known about their effect size, penetrance and genetic predisposition towards a certain phenotype, it is too early to use those rare CNVs for diagnoses of any NDs.

Among the aforementioned associated CNVs, this paper aimed to focus on the 1q21.1 CNV that is found with high incidence in ASD, SCZ, ADHD, ID and epilepsy [16]. Due to its structural complexity and inconsistent clinical phenotypes, this genetic locus has been understudied. A significant and popular finding in 1q21.1 is its mirror effect on neurodevelopment: microdeletions are widely found in the cases of SCZ, and microduplications are widely found in the cases of ASD [17]. This review will discuss the genetic structure of the chromosome 1q21.1 at the molecular and cellular levels and summarize clinical phenotypes associated with the genetic rearrangement.

## 2. Chromosomal Mapping and Genetic Pathway of 1q21.1

### 2.1. Chromosomal Structure

**Figure 1:**
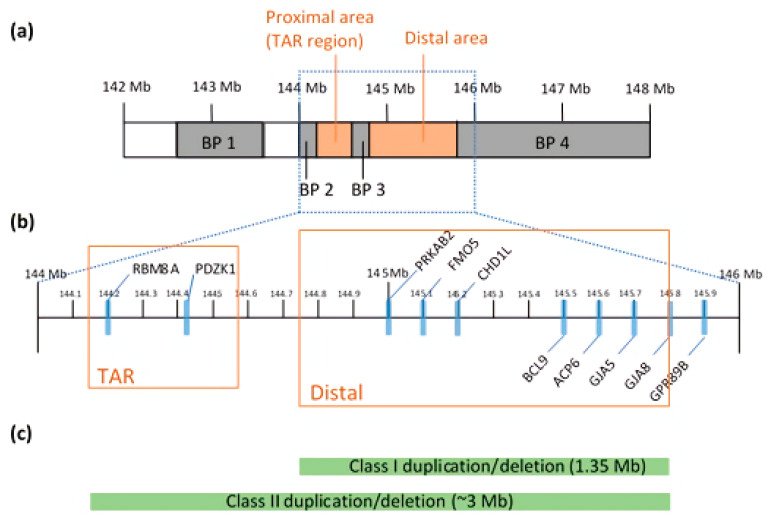
(**a**) Chromosomal structure of 1q21.1, mapped with four BPs (gray) and two distinct regions (red). (**b**) An enlargement of the region between 144 Mb and 146 Mb. Known genes commonly found in microduplication and microdeletion carriers are marked with blue bars. The reference locations on the chromosome are based on the March 2006 human reference sequence (NCBI build 36.1). The two distinct regions—TAR and Distal—are indicated by red blocks. (**c**) The two classes of duplications and deletions are shown with green bars. The size of the bars represents the minimally affected region in each class.

The 1q21.1 CNV is found within a 144 to 148 Mb region [18] (Figure 1a). In contrast to small CNVs, which are less detrimental, larger CNVs (>500 kb in size) can alter the expression levels of multiple genes [19]. It is a complex locus to study in that it not only spans 20–40 putative genes, but the region is also susceptible to genomic rearrangements due to the numbers of low copy repeats (LCRs). The more LCRs in the region, the more prone it is to frequent non-allelic homologous recombination (NAHR) during meiosis [20]. Clustered with LCRs, breakpoints (BPs) divide the locus into four possible segmental blocks and complicate the mapping and prediction of phenotypic expressivity [18]. Many of the LCRs and BPs are located adjacent to the crossing over points, making it difficult to estimate the phenotypes or genomic sequences in any given persons [21]. Through this mechanism, the CNVs, emerging in chromosomal duplications or deletions, can alter some of the dosage-sensitive genes and create a broad range of phenotypic variability [22]. Array comparative genomic hybridization and fluorescent in-situ hybridization analyses mapped out the overall structure of the 1q21.1 in great detail. The 1q21.1 region is associated with mental retardation, autism [23], schizophrenia [24] and microcephaly [21]. Duplication of 1q21.1 is strongly associated with autism [21]. 

Duplications and deletions are classified into two classes: Class I and Class II. Class I duplication/deletion involves only the distal 1q21.1 region between BP3 and BP4 (1.35 Mb in size), whereas Class II duplication/deletion extends from the distal 1q21.1 to the proximal 1q21.1 commonly detected between BP2 and BP4 (~3 Mb) [21] (Figure 1c). Combined data show enrichment in Class I deletions and duplications with a parental origin, but the components of genes and BPs can be varied after generations [25]. Both analyses discovered two distinct regions: proximal and distal 1q21.1, where a genomic gain or loss occurs (Figure 1b) [21,26]. Microdeletions at proximal 1q21.1 are mainly associated with thrombocytopenia-absent radius (TAR) syndrome and this region is often referred to as the TAR region. In particular, a core exon junction complex gene, *RBM8A*, is located in the TAR region and compound mutations in the *RBM8A* gene cause the TAR syndrome [27] that is comorbid with ID [28]. Other brain dysfunctions, including psychosis, agenesis of the corpus callosum and hypoplasia of the cerebellar vermis, are present in TAR patients [28,29,30]. Consistent with human patient studies, knockdown and knockout of *Rbm8a* in a mouse model revealed the critical role of *RBM8A* in neural progenitor cell (NPC) proliferation, neuronal migration and interneuron development, and loss of function in *RBM8A* in NPCs causes microcephaly [31,32,33]. Moreover, *RBM8A* plays a key role in adult neurogenesis and in regulating anxiety-related behavior [34], further supporting the important role of *RBM8A* in psychiatric diseases.

### 2.2. Genetic Architecture

**Figure 2:**
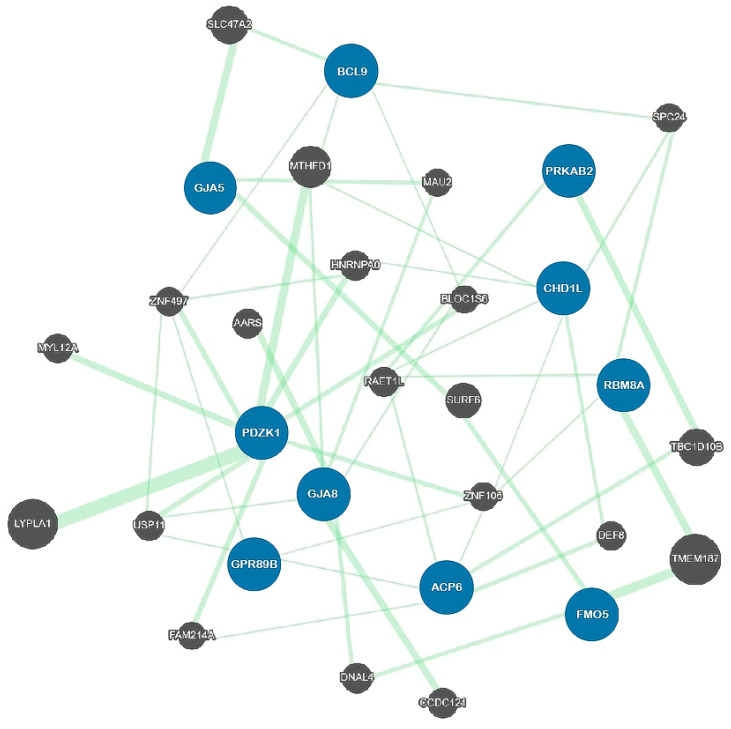
A genetic map of the associated genes observed in 1q21.1 microduplications and microdeletions. Blue circles are the 10 affected genes; black circles are the 20 related genes. None of the 10 core risk genes interacts directly with another. A total of 49 genetic linkages are drawn with different widths of green lines and were generated by the GeneMANIA program [42]. Gene expression of the candidate genes is positively correlated with the copy number of 1q21.1 but not with phenotypic severity. Even within the same genetic components, clinical presentations are shown to a different extent in cases, which denies the one gene–one phenotype module. The blue circles are the major genes discussed in the paper. Eight top-ranked genes in the correlation study are not directly linked to each other but are indirectly connected via subtype genes.

The recent advanced genomic assay has deciphered the genes encoded in the region and the position on the locus. The core genes commonly affected in the 1q21.1 CNV carriers are PRKAB2, FMO5, CHD1L, BCL9, ACP6, GJA5, GJA8, GPR89B and PDZK1 [25,35,36] (Figure 2; Table 1). However, the genetic study of the risk genes is far from clear as to the phenotypic consequences. Reported clinical phenotypes of the 1q21.1 duplication and deletion are not consistent, and no single gene has been confirmed to cause a pathologic effect in human studies [36].

This complex expressivity can be explained by a *cis*-epistasis genetic model. In contrast to a single gene CNV model, the gene expression is regulated by one or more CNV drivers and multiple modifiers [37]. Gain or loss of a single gene contributes only a small effect to trigger explicit clinical phenotypes [38]. This was confirmed in a number of genotype–phenotype association studies. A correlation analysis between gene expression and the copy number of 1q21.1 indicated that the candidate genes drew a positively correlated trend, in which a duplication CNV model was likely to have increased gene expression and vice versa [25], but the clinic severity may not have been correlated with the level of gene expression [39]. Harvard et al. conducted a family-based study of 1q21.1 microdeletion and microduplication and showed that individuals with the same CNV exhibited different levels of severity despite the identical gene components and almost identical BPs. Entangled chromatids are increased in lymphoblast cells derived from patients carrying both duplication and deletion of 1q21. To narrow down the causal gene, they identified two candidate genes, *CHD1L* and *PRKAB2*. Knockdown of *CHD1L* led to increased micronuclei in response to a topoisomerase II inhibitor, ICRF-193. However, both deletion and duplication carriers show the same cellular phenotype, suggesting that the gene dosage difference may not correlate with severity of symptoms. These findings once again emphasize the characteristic of the variable expressivity and the *cis*-epistasis model of the 1q21.1 CNVs [40,41]. Nevertheless, understanding of a linkage between genetic imbalance and apparent phenotypes is still incomplete.

**Table 1:** Genetic function and known phenotypes of dosage-sensitive genes associated with 1q21.1.

	Function ^1^	Molecular/Cellular Phenotypes	References
CHD1	Chromatin remodeling and DNA damage response	Impaired decatenation checkpoint activation	[25]
PRKAB2	AMPK regulatory subunit; maintaining energy homeostasis	Neurodegeneration; learning and memory impairment	[42]
GJA8	Gap junction protein; Connexin50	Cataracts; cardiac myopathy; increased risk of SCZ	[43,44]
GJA5	Gap junction protein; Connexin40	Cataracts; cardiac abnormalities	[18,45,46]
PDZK1	Ion transporter protein; regulates second messenger cascades	Increased risk of ASD and psychosis	[36]
GPR89B	Voltage dependent anion channel	Unknown	
BCL9	Wnt signaling pathway	Increased risk of SCZ	[47]
FMO5	Modulator of metabolic aging		[48,49]
ACP6	Histidine acid phosphatase protein	Unknown	

^1^ The Genecards Human Gene Database.

### 2.3. Pathogenesis of Proximal 1q21.1

These clinical manifestations are associated with the genomic segmental regions on 1q21.1. The frequency of the chromosomal abnormalities was highly skewed to distal regions compared with proximal regions. Minimal deletions in BP2-BP3, known as the TAR syndrome region, however, raised a question of whether this region is benign or pathogenic. The overall chromosomal abnormalities in the proximal region were less frequent than in the distal region. However, the relative enrichment of proximal 1q21.1 in microduplication, especially with a low ratio of de novo inheritance compared with the microdeletions, suggests that the proximal BP2–BP3 region is responsible for clinical microduplication aberrations and is mild enough to maintain fecundity [50,51]. Bearing in mind that developmental delay (DD) is a common history in microdeletions and microduplications, the genes within the proximal BP2–BP3 region account for cerebral development in addition to TAR syndrome [51]. On the other hand, even though the head size was a notable phenotype by dosage, head sizes between the proximal microdeletions and microduplications were not found to be discrete, suggesting that the genes in the proximal region are not sufficient or not responsible for microcephaly/macrocephaly [51]. These findings confirmed the pathogenicity of the proximal 1q21.1 region; this should be re-evaluated on a large scale to be supportive. 

## 3. Dosage Effect on Molecular and Clinical Phenotypes

### 3.1. Clinical Manifestation of 1q21.1

Carriers of the 1q21.1 duplication or deletion share some similar spectra of symptoms. Clinic presentations appearing to be mirrored could be due to the converging downstream pathways of chromosomal deletion and duplication [37]. Two major disorders in the spectrum are ASD and SCZ, which are associated with duplication and deletion of the 1q21.1 region, respectively. The complex symptoms of SCZ typically start in late adolescence or early adulthood and lead to a lifetime of treatment for SCZ patients. According to Diagnostic and Statistical Manual of Mental Disorders (DSM–5), they are generally divided into three categories: positive, negative and cognitive. The positive symptoms include hallucinations, delusions and disorganized thoughts. The negative symptoms include a reduction in, or lack of, motivation, affective response, verbal speech, attention and enjoyment. SCZ patients also suffer from cognitive impairment, which includes deficits in attention, language, memory and executive function. Cognitive impairment has been seen in people with SCZ before the onset of positive symptoms, and there is a moderate and appreciable decline throughout their lifetime [52]. Additionally, SCZ patients suffer from other comorbidities, including substance abuse [53,54,55,56,57,58]. The clinical presentations of ASDs show that the impaired social–communication functions and restricted, repetitive patterns of behavior can be detected in children with ASD at the age of 2–3 years. The clinical presentations are highly variable. Patients with ASD often have an increased risk of other neuropsychiatric symptoms, including anxiety [59], memory deficit [60], hyperactivity, aggression and epilepsy. ASD patients are often comorbid with ID.

The 1q21.1 CNVs have been widely reported in ND studies. Consistent with pleiotropic traits, this CNV is associated with other psychiatric disorders [22,61]. Published data on 1q21.1 CNVs carriers have shown psychiatric symptoms, including ADHD; ID; internalizing disorders such as depression, anxiety and bipolar disorder; and microcephaly/macrocephaly [18,25,35,62]. Non-neurologic syndromes such as congenital anomalies, cataracts and short stature are prevalent in probands with 1q21.1 CNV [50]. These features were not significantly distinct between deletions and duplications, but were distinct from controls [35]. Many comparison studies have attempted to validate these frequent diagnoses with the 1q21.1 CNVs.

**Figure 3:**
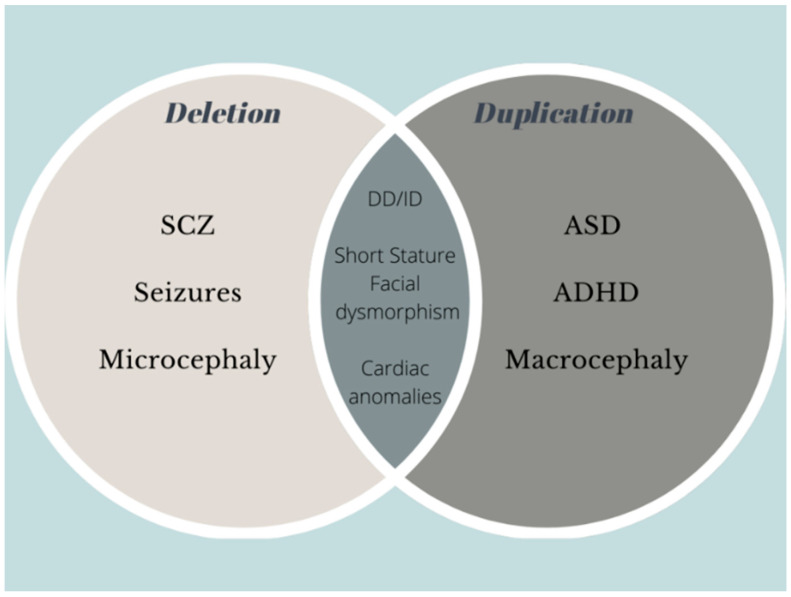
Venn diagram of phenotype association with 1q21.1 microdeletions and microduplication.

Case series studies have evidenced a phenotypic association with deletions and duplications. Microduplications show various and indefinite phenotypes, while deletions show a relatively consistent pattern [3]. Among neuropsychiatric disorders, cumulative literature ascertained that SCZ, microcephaly or relative microcephaly diagnoses were substantially higher in probands carrying microdeletions, and ASD, ADHD and macrocephaly or relative macrocephaly diagnoses were substantially higher in cases with microduplications [5,18,25,35,36,40,51,63,64]. Other recognizable syndromes also showed a distinguishing dosage effect, even though it was not constant, such as facial dysmorphism, heart and renal anomalies and behavioral problems (Figure 3, Table 2) [35,36,45,65]. 

### 3.2. Cognitive Impairment

The 1q21.1 CNVs show a strong association with developmental disabilities, including ASD [67], SCZ [8] and ADHD [64]. A large dataset indicates that individuals with developmental disabilities commonly accompany a history of cognitive deficits (Table 2). Most SCZ and ASD patients show cognitive impairment [6,7]. Larger CNVs, not limited to 1q21.1, led to a reduction of median IQ in a group of carriers [5], and SCZ-associated CNVs commonly confer a high risk of other mental disorders including ASD and ADHD, depending on the gene dosage [5,65]. Thus, the cognitive ability in patients is influenced by the risk CNVs (e.g., 1q21.1) during neurodevelopment, and cognitive deficit may contribute to pathophysiological outcomes in ASD, SCZ or ADHD. 

### 3.3. Head Size and Neural Abnormalities

Head size variation is another substantial phenotype mediated by genetic imbalance. It has been clearly replicated in many studies after the first clinical report of 1q21.1 examined the mean head circumference among carriers and found statistical significance (mean Z score for microdeletions: −2.55; microdeletion: +1.15; unpaired *t*-test, *p* < 0.0001) [21]. Recent studies focused on brain morphology with respect to gene dosage, since aberrant head shape during neurodevelopment may have disrupted the subsequent neuronal function, resulting in decreased axonal density. It has been hypothesized that head size abnormalities are driven by one of the candidate genes for neurological presentations such as neuronal differentiation and migration seen in 1q21.1 [36]. The copy number of the DUF1220 domain, encoded in the neuroblastoma breakpoint family (*NBPF*) gene family in 1q21.1, shows a strong correlation with brain size and neocortex volume [68]. This was supported by a case study of brain malformation [69]. The *NBPF* transcript level was significantly correlated with neuroblastoma susceptibility and it is highly expressed in the fetal brain, suggesting that the gene plays an important role in the developing brain [70]. Interestingly, a recent study collected brain images of SCZ patients carrying different CNVs, including 1q21.1, 15q11.2, 16p11.2, 17q12 and 22q11.2, and found no changes in whole brain volume but significant alterations in several midline white-matter structures [71]. Chromosome 1q21.1 itself is associated with brain size, as significantly decreased brain size was obtained with deletions (*p* < 0.001) [72]. Particularly, the microcephalic effect was predominant in the temporo-parietal, hippocampal, olfactory and subcortical regions, as well as the posterior midbrain regions [72]. This feature had the interhemispheric effect that the altered brain size was restricted strongly in the right hemisphere [73].

## 4. Molecular and Cellular Mechanisms Associated with 1q21.1 CNVs

### 4.1. Effect Range of 1q21.1 CNVs 

Studies of CNV pathogenesis have shown that deletions have deleterious effects, while duplications exhibit mild phenotypes [4]. Consistent with its pathogenicity, individuals with deletions have low fecundity and therefore undergo negative selection pressure [74,75]. These features of pathogenic CNVs appear in populations with low frequency and high mutation rates [8,74]. In light of this fact, it has become mainstream in genetic studies to distinguish distinct effect sizes in each ND. In line with the comparable burden of each structural variant, duplications exhibit a smaller burden than deletions in the synaptic pathway; functional clusters of duplications are enriched in NMDA receptor signaling, while functional clusters of deletions are enriched in the nervous system or behavioral phenotypes [15].

Examination of the cellular phenotype is a crucial step in the study of pathogenesis. Because many implicated risk genes in 1q21.1 CNVs are responsible for different cellular processes, including cell signaling, sensing and repair, impairment of these gene functions is expected to disrupt the cellular functions specifically involved with brain development and, in turn, to cause diseases [25]. However, a systematic pathological analysis of postmortem brains carrying 1q21.1 CNVs is still lacking. Due to the clinical manifestation reported among patients, animal models that mimic the genetic deficiency of 1q21.1 CNV could be good tools to provide some mechanistic insights and cellular and molecular targets for further therapeutic development [8,72,76]. 

### 4.2. Synaptic Signaling Pathway

Genes for cell signaling are enriched in 1q21.1 [67]. Cell signaling in the brain is impeded by abnormal synaptic plasticity. The dopamine hypothesis has been proposed in many ND studies, including ASD [77] and SCZ [78,79]. A 1q21.1 deletion mouse model recapitulated the function of 1q21.1 CNV in cellular phenotypes [76]. The 1q21.1 CNV accounts for the increased sensitivity to psychostimulants (e.g., amphetamine) and increased dopamine cell firing, and hypersensitivity is not mediated by a different number of D1/D2 receptors [76]. Thus, the findings are consistent with previous studies showing that 1q21.1 deletion shows a higher prevalence in SCZ patients than in ASD [78].

Alteration of the potassium channel function can impair in the whole neural network. Disruption of potassium ion homeostasis often becomes an initiator of the cells’ pathological cascade. In light of the crucial function of the potassium channel in neurodevelopment, GWAS has revealed a genetic overlap between rare risk CNVs (e.g., 1q21.1) and genes (e.g., *KCNN3*) encoding the potassium pump, transporter and channel [80,81,82]. The longer CAG repeats within the *KCNN3* gene seem to be associated with SCZ patients [81,82]. However, other studies did not confirm this association [83]. Interestingly, a mutant KCNN3 channel found in a SCZ patient was localized in the nucleus and inhibited the current mediated by another potassium channel, KCNN2 [84]. Therefore, the SCZ KCNN3 variant can function as a dominant-negative mutant to suppress endogenous small-conductance K currents and interfere with neuronal firing. Consistent with this notion, dysfunction in astrocyte differentiation derived from SCZ patient-derived induced pluripotent cells (iPSCs) was a result of excessive downregulation of potassium transporters in SCZ glia [80]. 

### 4.3. Mitochondrial Functions 

Mitochondrial diseases are often associated with ASD children [85]; as a result, creatine kinase, ammonia and aspartate aminotransferase have been used biomarkers for mitochondrial dysfunction in ASD [86]; however, the scale of these studies is still small. In animal studies, AMP-activated protein kinase (AMPK) function is modulated by one of the highest correlated genes, *PRKAB2* [25] in a *Drosophila* model of 1q21.1 [42]. A study confirmed that decreased AMPK activity impaired synaptic plasticity, which is critical for working memory and learning, and leads to sleep dysregulation and shortened lifespan [42]. Loss of AMPK activity also has been associated with the neurodegeneration phenotypes in a fly model of mitochondrial dysfunction [87]. Intriguingly, transcriptomic analyses of the three CNV mouse models—hemizygous deletions in corresponding regions of 1q21, 15q13 and 22q11—have identified that neuronal mitochondrial genes are consistently downregulated across three mutant genotypes and are shared with the transcriptomic changes observed in both SCZ and ASD postmortem brains [88]. This study suggests a previously understudied mitochondrial hypothesis underlying neuropsychiatric diseases associated with CNVs [89,90].

### 4.4. The WNT Signaling Pathway and BCL9 

Epidemiological studies have revealed that the prenatal period is vulnerable to ASD [91,92,93,94,95,96] and SCZ [97,98,99,100,101,102,103,104,105]. Among the key signaling pathways regulating fetal brain development, Wnt proteins play indispensable roles in angiogenesis [106,107,108,109,110], neurogenesis [111,112,113,114,115,116,117,118], cell survival [119,120,121,122], synaptogenesis [123,124,125] and neurite outgrowth [126,127]. The canonical pathway is well known to play a major role in neural development [128]. WNT signaling is regulated by several key components of the canonical Wnt pathway, including β-catenin, whose level determines the activity of canonical Wnt signaling. Recently, mutations in β-catenin have been identified as a frequent cause of ID (OMIM #615075), known as CTNNB1 syndrome [129,130,131,132,133,134], with some individuals also being diagnosed with ASD [135,136,137,138,139]. CTNNB1 syndrome patients are characterized by low IQ, microcephaly and facial dysmorphism that cannot be attributed to a known clinical syndrome [129,130,131,132,133,134]. A β-catenin conditional KO mouse specifically in PV interneurons showed that β-cat cKO mice have increased anxiety, impaired social interactions and elevated repetitive behaviors, which mimic some core symptoms of patients with ASD [140]. In addition, several mouse models with KO of Wnt regulators have shown consistent ASD-like behavioral deficits, including APC [141], DVL1 [142] and PTEN [143,144,145,146]. These data provide compelling evidence that an abnormal Wnt pathway is involved in the development of mental illness. 

The *BCL9* gene is located within the 1q21 region and encodes a nuclear retention factor for β-catenin, a critical part of the WNT signaling pathway [147,148,149]. BCL9 is essential for activation of the Wnt signaling in adult myogenic progenitors and regulates muscle regeneration [150]. To determine whether common variants in 1q21 can function as a candidate risk of SCZ, a large-scale GWAS comprising 5772 control and 4187 SCZ patients and 1135 patients with bipolar disorder was conducted in the Chinese Han population [47]. Interestingly, multiple SNPs within the *BCL9* gene are significantly associated with SCZ. Consistently, other GWAS and integrative analyses suggest that *BCL9* is associated with negative symptoms in SCZ [151,152] and is one of top risk genes in CNV [153]. As disruption of the BCL9–β-catenin interaction inhibits Wnt activation [154], which has been proposed as a therapeutic target for cancer [155,156], it remains to be tested if increasing BCL9 levels or fine-tuning WNT signaling could reverse the deficits caused by 1q21 CNV. In addition, several components of the Wnt signaling show an association with SCZ [157,158,159,160,161] and other psychiatric disorders [135,162,163]. Among the genetic factors associated with schizophrenia, the DISC1 [164] gene is a genetic risk factor for major mental illness [165,166,167,168,169]. DISC1 is a key regulator of NPC proliferation and mouse behavior through modulating the canonical Wnt signaling pathway [170]. DISC1 regulates cortical NPC proliferation and neuronal differentiation via inhibition of GSK3β. Treatment with pharmacological inhibitors of GSK3β can completely ameliorate the DISC1 loss-of-function-induced progenitor proliferation defects and behavioral abnormalities, which illustrates the exciting opportunity to develop small-molecule modulators of the Wnt pathway as prototypical drug treatments for psychiatric diseases. 

## 5. Discussion

Genetics analysis has become a powerful tool to investigate the etiology of psychiatric disorders because many NDs show high inheritance and share a strong genetic correlation [61]. The CNV burden is pronounced in affected individuals [16,171]. However, the rare (<1% frequency) and large (>500 kbp) CNVs in the 1q21.1 region are also found in unaffected individuals, which raises the question of whether microdeletions and microduplications of 1q21.1 are benign. Accumulative data showed that these variants were enriched in affected cases compared with unaffected ones, supporting that these variants contribute to the risk of disease [18]. However, the pathogenic CNVs were found with inconsistent patterns of inheritance and clinical phenotypes in family studies, which complicates predictions of genotype–phenotype correlations [10]. Nonetheless, active analyses of the genotype–phenotype association have revealed some possible linkages; for example, GJA5 and cardiac phenotypes [18], neurexins/neuroligins and synaptic differentiation [66], or ROBO1 and dyslexia [172].

As 1q21.1 CNV is strongly associated with developmental disabilities, the symptoms are severe in childhood, and the affecting range of 1q21.1 is different between adults and children [35,63]. However, a gap remains to compare severity in childhood (<18-year-olds) and adulthood (>18-year-olds) within the same populations. Along with the general tendency of mild duplication phenotypes, there might be ascertainment bias in the clinical reports, in which mild duplications might have been overlooked or not been apparent in carriers at the moment of collecting data. Moreover, a possibility that mild psychiatric symptoms may have a late onset is another confounding factor. For a precise assessment, psychiatric evaluations in populations should be taken for the long term. The comparison studies reported that phonological processing and fine motor performance were significantly lower in microdeletion and microduplication probands considering only children and compared with adults [35,173]. This combined finding was confirmed in a 1q21.1 microduplication child with severe language and motor ability delay [172]. Besides the developmental stage, sex bias should be eliminated in sample collections for ND studies, especially ASD [174].

Profound and solid evidence with large controls and samples is required to refine the current findings. However, owing to the rarity of pathogenic CNVs in populations, current studies suffer from the limitation of acquiring a sufficient number of patients for each CNV type. This review discusses the specific CNV 1q21.1 as an important contributor in many psychiatric diseases.

## 6. Conclusions

There exist several limitations that hamper the validation of the GWAS findings. In order to investigate the etiology of a genetic disease, (i) a large sample size and (ii) examination of the molecular mechanism are required. The collective data, which include detailed clinical manifestations and genetic variations of individuals, like the type and number of CNVs as well as medical history, can consolidate observations of unique phenotypic patterns related to a certain genotype. However, there has been a difficulty obtain extensive pedigrees of probands of the disease due to its scarcity. It is unfortunate that a lot of research has been based on a limited sample size, usually less than 1000 patients. Along with clinical observations, verifying the molecular mechanism underlying the disease is critical. Animal models have come to be a useful tool, as they facilitate molecular observations that are impossible to assess directly from the human brain. However, the animal models do not fully satisfy the full extent of diagnostic symptoms in humans such as cognitive and internalizing symptoms. By the same token, there have not been sufficient animal models tested, owing to the difficulty of generating the replicated genetic rearrangements, especially 1q21.1 microduplications and neuropsychiatric symptoms in animals [175,176]. This can be complemented by using iPSCs [177]. Moreover, as most psychiatric disorders are polygenic, it is difficulty to declare that 1q21.1 CNVs are the single contributor of such phenotypes. In addition to the genetic factors, another pronounced contributor is the environment. Several studies have postulated that stress in early development and methylation can alter gene expression, producing an increased risk of develop the disease [178]. Moreover, even geographic exposure was found to be a factor among global populations [14].

Recently, ND research has moved to investigating the interaction between genetic variants and environmental factors and to find genetic convergence across the NDs [12]. A new direction is the association of neuroinflammation with NDs. A significant upregulation of microglial expression is observed in ASD and SCZ [179], and an imbalance of microglial activation is present with psychotic symptoms [180], suggesting another possible underlying mechanism [181]. As an emerging field in NDs [182], how neuroinflammation affects the penetrance and risk of rare CNVs in the diseases should be assessed in future studies. 

## Figures and Tables

**Table 2:** Case reports of neurodevelopmental diseases with 1q21.1 duplications and deletions.

References	Duplication (2nd CNV Cases)						Deletion (2nd CNV Cases)						Total Cases
	ASD	SCZ	ID	ADHD	TAR	Other DD	ASD	SCZ	ID	ADHD	TAR	Other DD	
[35]	7		5	5		4	2		2	1		3	29
[21]	3			1		13	1			2		16	36
[18]	4					5 (1)	2			2		16 (2)	32
[51]	3			5		11	4		1	3	4	25 (5)	61
[50]	3		3	3		2			5	1	1	4	22
[66]	3 (1)						1						5
[13]		8						10 (2)					20
[8]		1						4					5
[9]								10					10

All reported cases are single CNV carriers except for the 2nd CNV carriers, which are stated within parentheses. All cases include de novo and inherited CNVs. A blank cell indicates that the data are not available.

## Data Availability

Not applicable.

## Abbreviations

ADHD	Attention deficit hyperactivity disorder
ASD	Autism spectrum disorder
BP	Breakpoint
CNV	Copy number variation
DD	Developmental delay
GWAS	Genome-wide association study
ID	Intellectual disorder
LCR	Low copy repeats
NAHR	Non-allelic homologous recombination
ND	Neurodevelopmental/neuropsychiatric disorder
SCZ	Schizophrenia
TAR	Thrombocytopenia-absent radius
NBPF	Neuroblastoma breakpoint family
NPC	Neural progenitor cell
KO	Knockout
PV	Parvalbumin
iPSCs	Induced pluripotent cells
